# Supplementary material for: Evidence for G6PD variant classification from multiplexed functional assays
Source: Genome Biol. 2026 Jun 4;27:249. doi: 10.1186/s13059-026-04105-y (PMC13455394; doi:10.1186/s13059-026-04105-y)
Supplement: Supplementary file 1 [file 13059_2026_4105_MOESM2_ESM.docx]

**Additional File 5: Supplementary Figures S1-S6.**

**Fig S1. Variants of known effect in G6PD activity MAVE.** (A) Frequency of reference and G6PD variants that do not lead to deficiency and (B) that do cause deficiency within the selection turbidostats over time. Activity scores for each variant presented in the panel. Shapes denote replicates; activity in red blood cells (RBCs) from literature reports.

**Fig S2. G6PD activity MAVE.** (A) Population doublings in the turbidostats, calculated from pump time. Shapes denote replicates. (B) Normalized activity score correlation between replicates. (C) Distribution of variants by normalized activity scores. (D) Correlation between reported variant activity in red blood cells and MAVE activity score, p<10^-6^. (E) Frequency of each variant over time in selection. Line represents linear fit to both selection replicates.

**Fig S3. G6PD abundance MAVE.** (A) Normalized abundance score correlation between replicates. (B) Distribution of variants by normalized abundance scores.

**Fig S4. G6PD substrate and dimer interactions.** (A) Scores mapped onto G6PD-D200N tetramer bound to NADP^+^ and G6P (7SNI), highlighting substrates G6P and NADP^+^ and (B) structural NADP^+^ and the dimer interface. Dimer interface panel highlights one G6PD molecule in the dimer in pink, and the other in gray.

**Fig S5. Comparison of functional scores and variant effect predictors.** (A) *G6PD* variant functional scores compared to scores from VEPs REVEL and (B) Alpha Missense. Significance from by ANOVA with Tukey’s HSD, only showing statistics for difference compared to intermediate or ambiguous class; ****, p<0.0001. (C) *G6PD* missense variant scores from REVEL (n=3,428) and (B) Alpha Missense (n=10,127); dashed lines represent score calibrations and classes as in A-B.

**Fig S6. Benchmarking of abundance and activity scores.** (A) Abundance scores and (B) activity scores of variants classified on ClinVar as known pathogenic / likely pathogenic and benign / likely benign. Lines denote z-scores of -1 and -3 from the synonymous distribution (0.8814 and 0.6421 for abundance; 0.9130 and 0.7657 for activity), and Odds of Pathogenicity (OddsPath) was calculated with those cutoffs as described for functional scores.
